# Three-Electrode Electrochromic Energy Storage Devices with Enhanced Multicolor Functionality

**DOI:** 10.3390/mi17091020

**Published:** 2026-08-27

**Authors:** Ming-Yue Tan, Pei-Ling Low, Gregory Soon How Thien, Kar Ban Tan, Yun Ii Go, Jingsong Huang, Kah-Yoong Chan

**Affiliations:** 1 Faculty of Artificial Intelligence and Engineering, Multimedia University, Persiaran Multimedia, Cyberjaya 63100, Malaysia; 2 Centre for Advanced Devices and Systems, COE for Robotics and Sensing Technologies, Multimedia University, Persiaran Multimedia, Cyberjaya 63100, Malaysia; 3 Department of Chemistry, Faculty of Science, Universiti Putra Malaysia, Serdang 43400, Malaysia; 4 School of Engineering and Physical Sciences, Heriot-Watt University Malaysia, 1, Jalan Venna P5/2, Precinct 5, Putrajaya 62200, Malaysia; y.go@hw.ac.uk; 5 Oxford Suzhou Centre for Advanced Research (OSCAR), University of Oxford, Suzhou 215123, China

**Keywords:** electrochromic, multicolor, energy storage

## Abstract

Electrochromic (EC) technology has garnered attention for its low energy consumption and aesthetic versatility. Among various EC materials, tungsten trioxide (WO_3_) is notable for its high optical contrast, durability, and coloration efficiency. However, its single-color output limits its versatility in applications such as multicolor displays and windows. In addition, conventional complementary EC devices require the pairing of anodic and cathodic materials, restricting independent control of redox reactions on the electrodes. Hence, this study introduced a three-electrode EC energy storage device with various configurations: vanadium oxide/zinc/vanadium oxide (V_2_O_5_/Zn/V_2_O_5_) (energy status indicator) and V_2_O_5_/Zn/WO_3_ (energy storage window and multicolor display). This design allowed independent redox control on both electrodes, enhanced multicolor functionality through V_2_O_5_, and incorporated zinc metal for efficient energy storage and discharge-induced coloration. The study revealed that V_2_O_5_/Zn/V_2_O_5_ configuration exhibited higher coloration depth (58.92% for orange, 32% for yellow-green, and 10.65% for blue) across all color states while achieving a high energy storage capacity of 185 mAh/m^2^, making it ideal for energy status indicator applications. Conversely, the V_2_O_5_/Zn/WO_3_ structure achieved a significantly higher optical contrast of 77.86%, excellent coloration efficiency of 46.24 cm^2^/C, and energy storage capacities of 104.8 mAh/m^2^ (V_2_O_5_) and 142 mAh/m^2^ (WO_3_), making it suitable for multicolor energy storage windows. Additionally, the V_2_O_5_/Zn/WO_3_ configuration demonstrated suitability for multicolor display applications. Overall, these three-electrode EC energy storage architectures address the intrinsic color limitations and redox control constraints of conventional devices, enabling high-capacity, tunable multicolor operation with significant potential for integration into advanced smart windows, energy indicators, and adaptive display technologies.

## 1. Introduction

Electrochromic (EC) technology has garnered attention due to its low energy consumption and aesthetic versatility, enabling applications in energy-efficient smart windows, displays, and interior design [1]. EC devices typically consist of an EC thin film and an electrolyte, allowing the device to change its optical properties (transparent, translucent, or opaque) in response to an applied voltage [2]. This optical transformation is driven by the movement of ions between the electrolyte and the EC thin film: the insertion of ions into the film induces a color change, while their extraction reverts the thin film to its original optical state.

Among the various EC materials, tungsten trioxide (WO_3_) stands out for its high optical contrast, durability, and coloration efficiency [3,4,5]. However, WO_3_-based devices are generally limited to a single color, most often a monotonous dark blue [6]. This lack of color versatility constrains their broader aesthetic applications, such as in multicolor displays and architectural designs. In contrast, vanadium pentoxide (V_2_O_5_) has attracted considerable interest for multicolor electrochromism as reversible changes in its oxidation state can produce multiple color states [7,8].

Additionally, conventional EC devices are typically restricted to a two-electrode configuration, in which one electrode must be anodic and the other cathodic, a design commonly referred to as a complementary EC device [9,10]. This configuration restricts control over the redox reactions, as the reduction on one electrode is inherently coupled to oxidation on the other [11]. In addition, Ke et al. reported that an imbalance in the charge capacities between the cathode and anode led to non-uniform coloration in the complementary EC device [12]. Such limitations reduce the flexibility of color states and hinder customization for diverse applications. In parallel, sustainable electrode materials have been increasingly explored for emerging energy storage systems. For instance, hard carbon has been investigated as a promising anode for sodium-ion batteries [13], while Zn and Al have been employed as active electrodes in EC energy storage devices owing to their abundance, high theoretical specific capacity, and low-cost properties [14,15]. In particular, Zhang et al. demonstrated a Zn-based EC configuration by constructing an EC display with a Zn layer positioned between two SVO (sodium vanadium oxide) electrodes [11]. The device exhibited reversible switching among multiple colors, including orange, amber, yellow, brown, chartreuse, and green, while maintaining high optical transparency.

In our previous study, a Zn^2+^/Al^3+^ water-in-bisalt (WiBS) electrolyte was optimized and developed for Zn-based WO_3_ energy storage devices, demonstrating favorable optical modulation and energy-storage capability [16]. Building upon this established electrolyte system, the present study extends its application to a three-electrode EC energy-storage architecture comprising two distinct configurations: vanadium oxide/zinc/vanadium oxide (V_2_O_5_/Zn/V_2_O_5_) (energy status indicator) and V_2_O_5_/Zn/WO_3_ (energy storage window and multicolor display). This structure addresses the limitations of conventional EC devices and offers several advantages. First, the three-electrode design eliminates the conventional requirement of pairing anodic and cathodic EC materials in a single device. This design allows users to independently control the redox reactions and the corresponding color changes on both electrodes based on their needs. For instance, both electrodes can be adjusted to appear transparent, opaque, or any combination thereof [17]. Second, the incorporation of zinc metal between the EC thin films imparts energy storage functionality by facilitating reversible charge transfer during operation. Third, the use of V_2_O_5_ as one of the EC materials introduces multicolor functionality. V_2_O_5_ can overlay and intensify the generated colors when paired with WO_3_, creating a richer color during the coloring state.

Notably, the thin films in all configurations were fabricated using the sol–gel spin-coating technique, a cost-effective and simple deposition method [4]. This study investigated the EC and electrical properties of the proposed devices, emphasizing their advantages and potential applications across various domains, including energy status indicators, window-integrated energy storage systems, and multicolor displays.

## 2. Materials and Methods

### 2.1. V_2_O_5_ Thin Film Preparation

All chemicals used in this study were of analytical grade and sourced from Sigma-Aldrich, Rockville, MD, USA. The fabrication of V_2_O_5_ sol–gel involved using vanadium (V) powder as the precursor and hydrogen peroxide (H_2_O_2_) as the strong oxidizing agent.

The sol–gel preparation process began by transferring 0.5 g of V_2_O_5_ powder into 30 mL of H_2_O_2_ solution. The mixture was stirred immediately at high speed, causing an exothermic reaction that transformed the solution into a viscous, dark red liquid. Once cooled, the sol–gel was transferred to a glass vial and aged for 24 h [18].

Before depositing the thin film, the indium tin oxide (ITO) glass substrates were thoroughly cleaned using an ultrasonic bath in ethanol, acetone, and distilled water, each for 10 min. For the deposition, the spin-coating method was used to deposit the V_2_O_5_ sol–gel onto the ITO glass substrate at a rotation speed of 3000 rpm for 30 s. The freshly coated layer was then heated on a hot plate at 100 °C for 3 min to evaporate the solvent. Finally, the V_2_O_5_ films with an approximate thickness of 280 nm were annealed in a furnace at 200 °C for 1 h.

### 2.2. WO_3_ Thin Film Preparation

The WO_3_ sol–gel fabrication process was carried out using tungsten hexachloride (WCl_6_) as the precursor, glacial acetic acid (CH_3_COOH) as a chelating agent, hydrogen peroxide (H_2_O_2_) as an oxidizing agent, and absolute ethanol (C_2_H_6_O) as the solvent.

To prepare the sol–gel, 1 g of WCl_6_ powder was weighed and transferred to a beaker. Subsequently, 20 mL of ethanol and 2 mL of glacial acetic acid were added to the beaker, and the mixture was stirred at 200 rpm until a dark blue solution was formed. Next, 2 mL of H_2_O_2_ solution was added, and the mixture was stirred continuously for 90 min. The resulting WO_3_ sol–gel was aged for 72 h before the deposition process. The deposition of the WO_3_ sol–gel followed a process similar to that used for V_2_O_5_, and the films were subsequently annealed in a furnace at 100 °C for 1 h. Notably, the obtained WO_3_ thickness was approximately 280 nm.

### 2.3. Metal Salt Electrolyte Preparation

The metal salt electrolyte was prepared by dissolving 0.03 mol of AlCl_3_ and 0.1 mol of ZnCl_2_ in 10 mL of distilled water. The mixture was stirred thoroughly to ensure uniformity, resulting in solutions with concentrations of 10 M ZnCl_2_ and 3 M AlCl_3_.

### 2.4. Device Assembly and Working Principles

**Figure 1:**
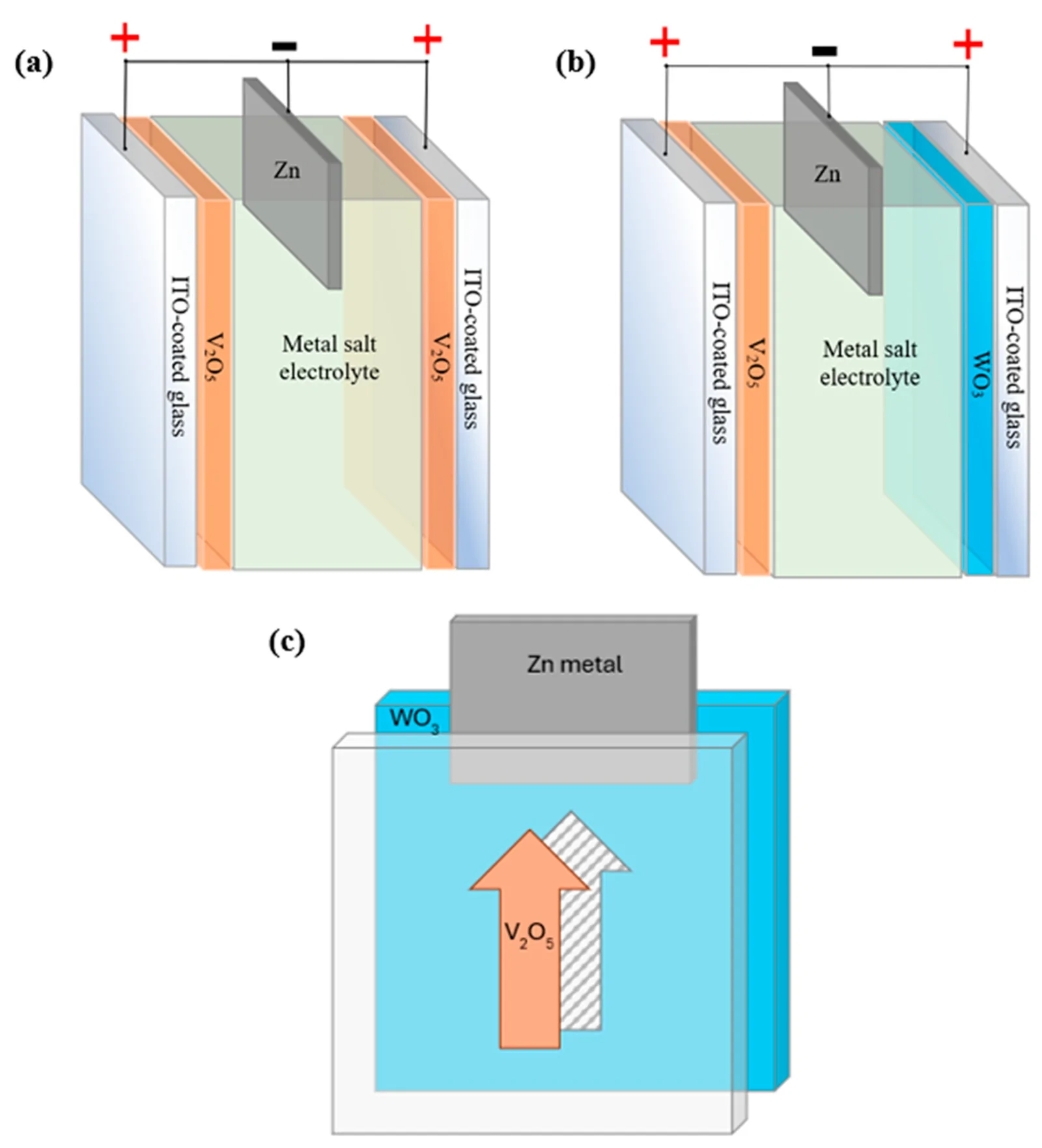
Three-electrode EC energy storage device structure: (**a**) V_2_O_5_/Zn/V_2_O_5_ (energy status indicator), (**b**) V_2_O_5_/Zn/WO_3_ (energy storage window), and (**c**) V_2_O_5_/Zn/WO_3_ (multicolor display).

The structures of the three-electrode EC energy storage devices: V_2_O_5_/Zn/V_2_O_5_ (energy status indicator) and V_2_O_5_/Zn/WO_3_ (energy storage window and multicolor display) are illustrated in Figure 1a, b and c, respectively. To obtain V_2_O_5_/Zn/V_2_O_5_ (energy status indicator) and V_2_O_5_/Zn/WO_3_ (energy storage window) devices, two EC thin films (either V_2_O_5_ or WO_3_) that were previously deposited onto ITO glass substrates were assembled in a parallel configuration, with a Zn plate and spacer sandwiched between them. Lastly, the electrolyte was injected into the devices and sealed with UV resin. The active area for both devices was 2.4 cm^2^.

**Figure 2:**
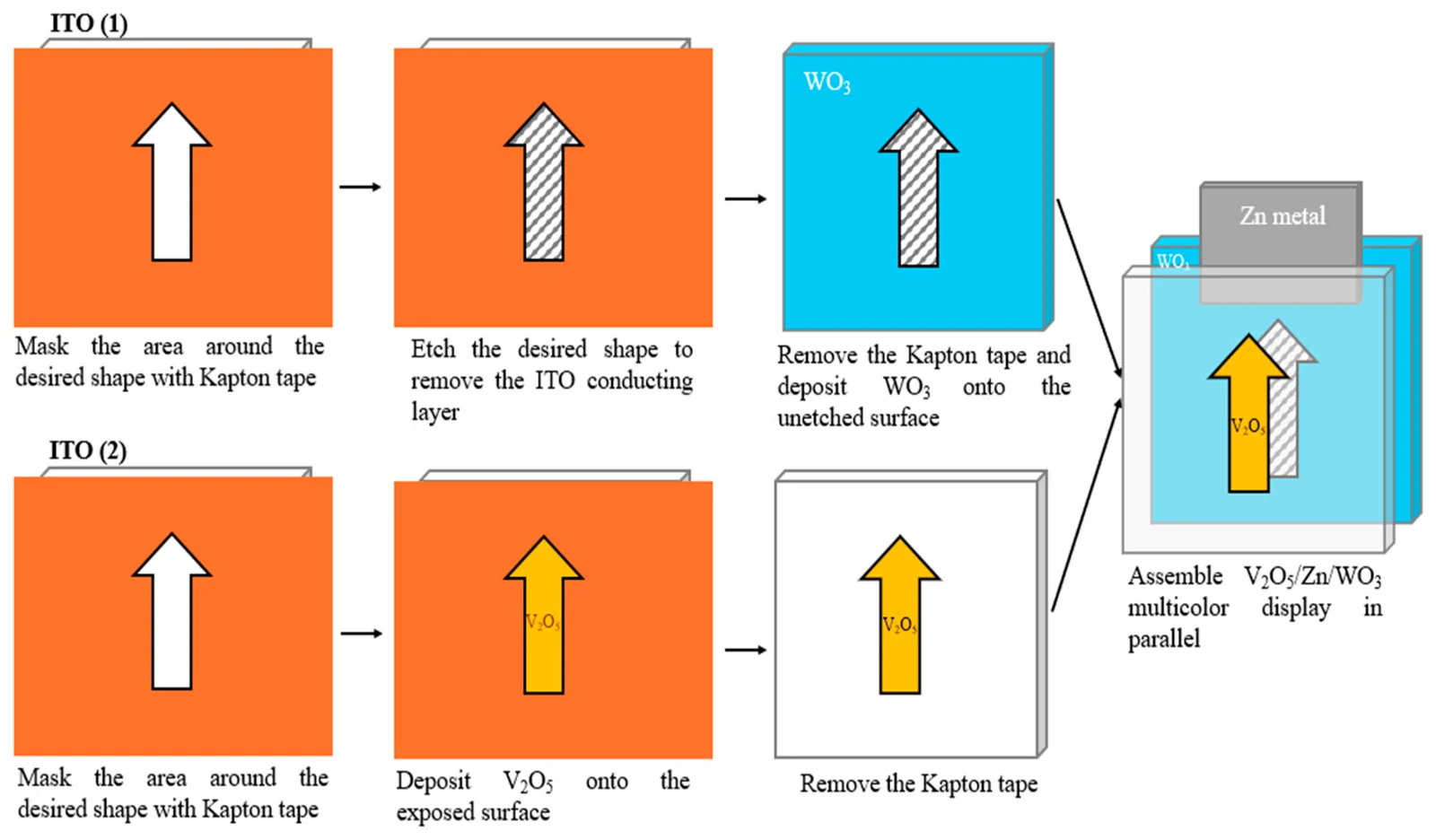
The fabrication process of a multicolor display.

Meanwhile, to fabricate the V_2_O_5_/Zn/WO_3_ multicolor display, two pieces of ITO-coated glass with dimensions of 5 × 5 cm were prepared. Firstly, the ITO glass (labeled ITO 1) was masked with Kapton tape, and a pen knife was used to craft the desired pattern. The exposed ITO areas were then etched to remove the conductive layer. After etching, the Kapton tape was removed, and WO_3_ thin films were deposited onto the unetched area. The second piece of ITO glass (labeled ITO 2) underwent a similar masking and patterning process, followed by the deposition of V_2_O_5_ thin films onto the exposed areas. Once the deposition was complete, the Kapton tape was removed. Finally, a zinc metal plate and electrolyte were sandwiched between the WO_3_-coated and V_2_O_5_-coated glass pieces and were sealed using UV resin. The fabrication process of the multicolor display is illustrated in Figure 2.

Typically, the three-electrode EC configuration consists of a working electrode (EC layer such as WO_3_ or V_2_O_5_) and a metallic Zn electrode functioning as the anode. In the discharging (coloring) process, Zn metal undergoes oxidation at the anode (Zn → Zn^2+^ + 2e^−^), releasing Zn^2+^ ions into the electrolyte. These ions migrate toward the EC electrode, where they participate in the reduction of transition metal cations (W^6+^ → W^5+^ or V^5+^ → V^4+^), accompanied by a corresponding color change, as reported in previous studies [16,19]. Meanwhile, the released electrons flow through the external circuit, which can simultaneously power external low-energy devices. Conversely, during the charging (bleaching) process, an external potential is applied to reverse these reactions. The intercalated ions are deinserted from the EC layers, and Zn^2+^ ions are reduced and electrodeposited back onto the Zn electrode (Zn^2+^ + 2e^−^ → Zn). In this process, the device enables energy storage by generating electrochemical potential energy within itself [20,21].

### 2.5. Characterization Techniques

The electrical characteristics of the EC energy storage devices were evaluated through galvanostatic charge/discharge (GCD) measurements using a Gamry Interface 1010E Potentiostat (Gamry Instruments, Warminster, PA, USA). Additionally, their optical transmittance was analyzed with a UV-Vis spectrophotometer (Avantes AvaSpec-2048L, Avantes, Apeldoorn, The Netherlands). Finally, the electrochromic performance was assessed using chronoamperometry (CA) and cyclic voltammetry (CV) techniques, performed with an Autolab PGSTAT204 system (Metrohm, Herisau, Switzerland).

## 3. Results and Discussion

### 3.1. Energy Status Indicator (V_2_O_5_/Zn/V_2_O_5_)

**Figure 3:**
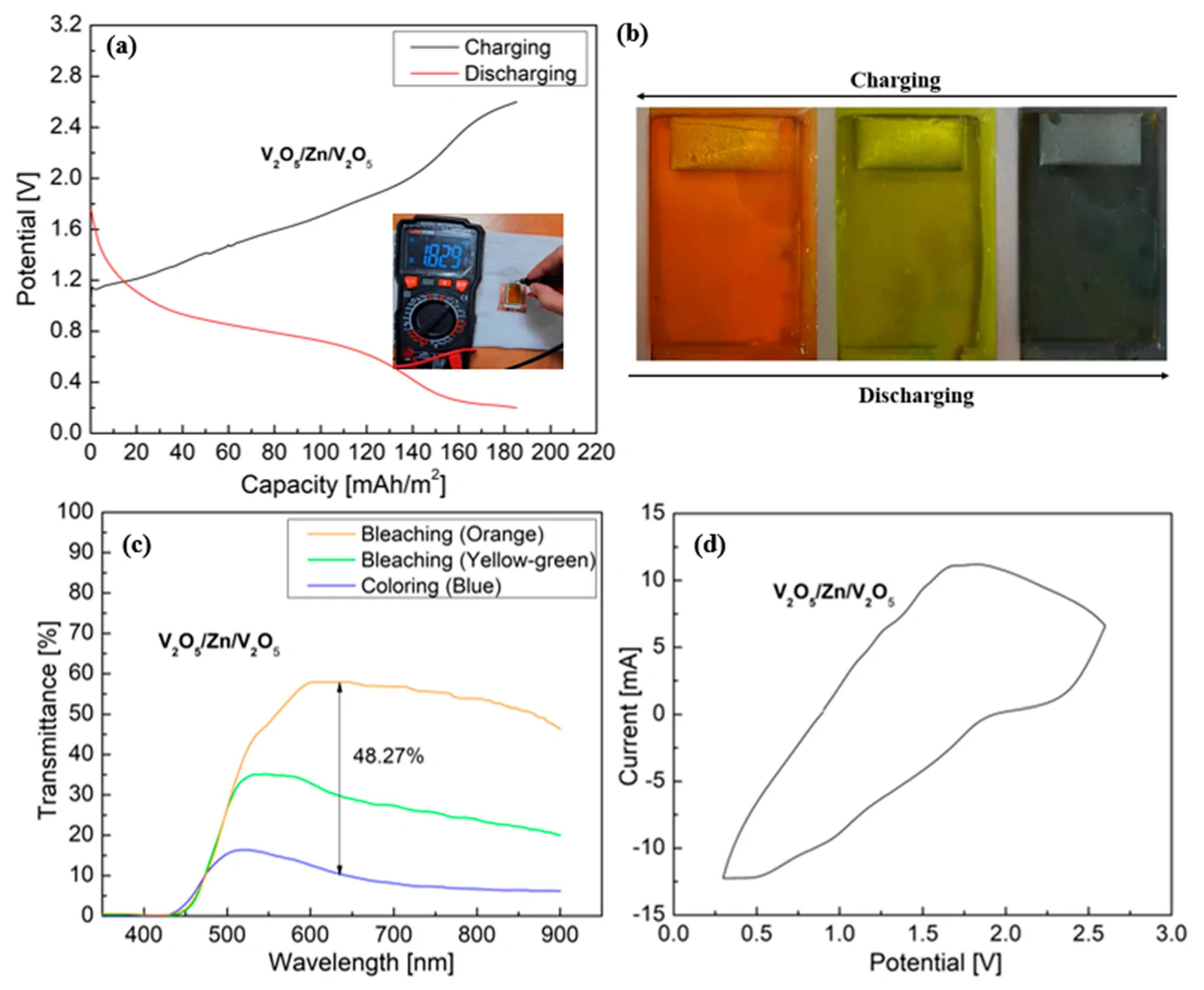
(**a**) Galvanostatic charge/discharge curves and open-circuit voltage, (**b**) color transitions at different energy charge levels, (**c**) optical modulation, and (**d**) cyclic voltammetry curves of the V_2_O_5_/Zn/V_2_O_5_ device.

Figure 3a illustrates the galvanostatic charge/discharge (GCD) curve of the V_2_O_5_/Zn/V_2_O_5_ device at a current density of 1 mA/cm^2^. The device exhibited an energy storage capacity of 185 mAh/m^2^, coulombic efficiency of 99.8%, and an open-circuit voltage of 1.8 V. Considering that most wireless sensor nodes and small LED indicators operate at 1.5–3.3 V with power consumption typically below 200 mW, the obtained voltage and capacity indicate that the device can provide sufficient energy for such low-energy systems. Moreover, Figure 3b illustrates the color transitions corresponding to different energy charge levels in the V_2_O_5_/Zn/V_2_O_5_ device. The fully charged device exhibited a dark orange color, which gradually changed to yellow-green and subsequently to dark blue as the device was discharged. These distinct color states provide a simple visual indication of the device’s charge status, demonstrating its potential for energy-status indication without requiring additional electronic displays or circuits.

Furthermore, Figure 3c reveals the optical transmittance at 633 nm for the V_2_O_5_/Zn/V_2_O_5_ device, measured at 58.92% for orange, 32% for yellow-green, and 10.65% for blue, yielding an overall optical contrast of 48.27%. The relatively low optical transmittance was due to the color overlay effect from the dual-sided V_2_O_5_ thin films. This effect resulted in greater coloration depths across the different color states, producing clearly distinguishable colors. These distinct color variations enabled the color states to be easily differentiated, which was advantageous for energy status indicator applications, where the power level needed to be readily identified. Additionally, Figure 3d presents the cyclic voltammetry (CV) graph, obtained by simultaneously applying a voltage range of +0.3 to +2.6 V to both sides of the V_2_O_5_ thin film at a scan rate of 0.1 V/s. The CV analysis revealed a high cathodic peak current of −12.8 mA, attributed to enhanced ion kinetics and increased active sites from the dual EC thin film configuration.

### 3.2. Multicolor Energy Storage Window (V_2_O_5_/Zn/WO_3_)

**Figure 4:**
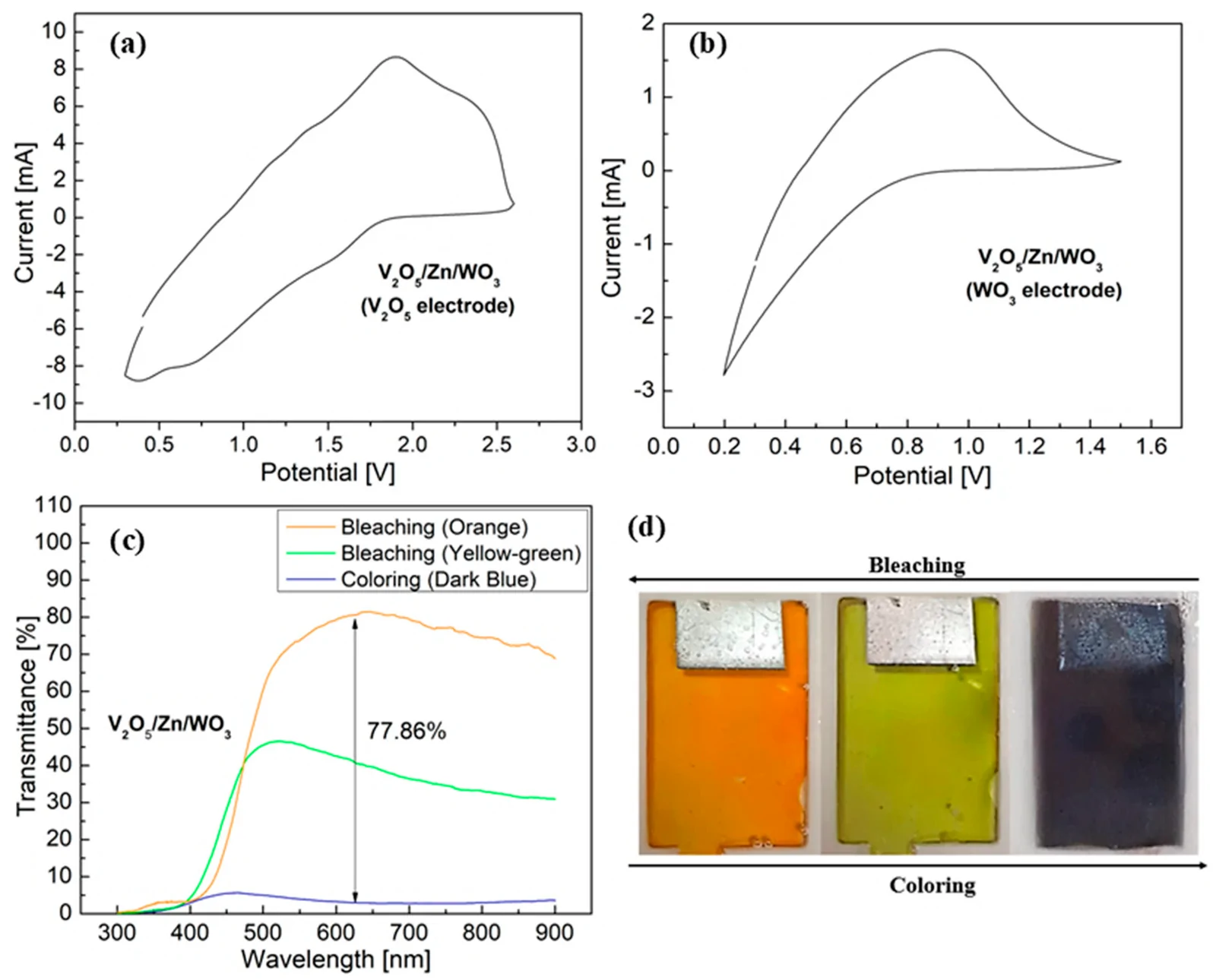
(**a**,**b**) Cyclic voltammetry curves of the V_2_O_5_ and WO_3_ electrodes, (**c**) Optical modulation performance, and (**d**) color transitions of the V_2_O_5_/Zn/WO_3_ device.

To explore the EC properties of the V_2_O_5_/Zn/WO_3_ device, proposed as a multicolor energy storage window, CV and CA measurements were conducted. Notably, the same voltage window was applied for both CV and CA measurements to ensure consistent electrochemical evaluation. Due to the use of two distinct EC electrodes with different redox potentials, separate voltage windows were applied to ensure optimal electrochemical activity. Specifically, a voltage range of +0.2 to +1.5 V was applied to the WO_3_ electrode, while +0.3 to +2.6 V was applied to the V_2_O_5_ electrode, both at a scan rate of 0.1 V/s. Figure 4a,b demonstrate cathodic peaks for the V_2_O_5_ and WO_3_ electrodes, with peak currents of −8.8 mA and −2.8 mA, respectively, corresponding to the electrochemical reduction and ion-insertion processes during coloration. In addition, as shown in Figure 4c, the device demonstrates high transmittance during the bleaching state, with 80.86% transmittance when transitioning to orange and 43% at yellow-green. Notably, during bleaching, the WO_3_ thin film remained transparent, while only the V_2_O_5_ thin film underwent color changes. In contrast, during coloring, both WO_3_ and V_2_O_5_ thin films transitioned to blue, resulting in a low transmittance of 3% due to the overlapping blue color from both electrodes. The color transitions of the V_2_O_5_/Zn/WO_3_ device can be observed in Figure 4d. Notably, compared to the V_2_O_5_/Zn/V_2_O_5_ configuration, which achieved an optical contrast of 48.27%, the V_2_O_5_/Zn/WO_3_ structure exhibited a significantly higher optical contrast of 77.86%, making it more suitable for window applications. Furthermore, the device exhibited moderate response times of 33 s for coloring and 19 s for bleaching, along with a high coloration efficiency of 46.24 cm^2^/C, highlighting its excellent EC performance. Notably, the switching times were determined based on 90% of the full transmittance modulation at 633 nm.

**Figure 5:**
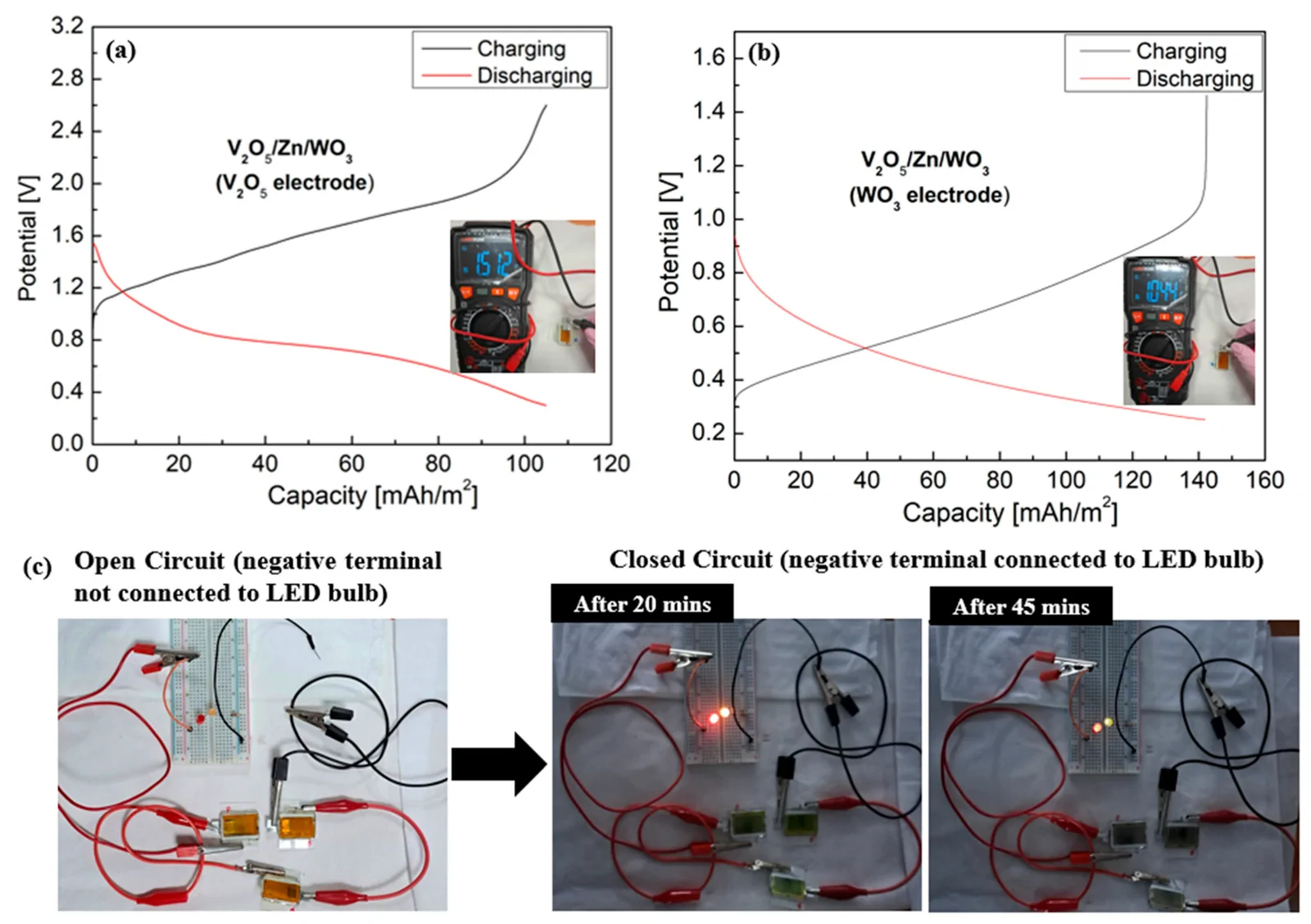
Galvanostatic charge/discharge curves and open-circuit voltage of the (**a**) V_2_O_5_ and (**b**) WO_3_ electrodes, and (**c**) demonstration of LED illumination driven by V_2_O_5_/Zn/WO_3_ devices.

Moreover, the V_2_O_5_ and WO_3_ electrodes demonstrated energy storage capacities of 104.8 mAh/m^2^ and 142 mAh/m^2^, respectively, with coulombic efficiencies of 99.8% and 99.7%, and open-circuit voltages of 1.5 and 1.0 V, respectively [see Figure 5a,b]. Similar to the V_2_O_5_/Zn/V_2_O_5_ device, the V_2_O_5_/Zn/WO_3_ structure is suitable for powering low-energy devices while offering aesthetic benefits. An example of this structure is a multicolor energy storage window integrated into homes or specialized buildings, such as hospitals and themed cafes. During the day, the window could display specific colors by applying different voltages (e.g., approximately 2.6 V for orange and 1.5 V for yellow-green) while simultaneously storing energy. In the evening or at night, the stored energy could be utilized to power LED or motion sensors, providing illumination and enhancing security. As the device discharges, it gradually transitions to a dark blue color, driven solely by its open-circuit potential, without requiring any external voltage—a phenomenon known as discharge-induced coloration. Figure 5c demonstrates that connecting three V_2_O_5_/Zn/WO_3_ devices in series could light up the LEDs.

### 3.3. Multicolor Display (V_2_O_5_/Zn/WO_3_)

The third proposed application utilized the V_2_O_5_/Zn/WO_3_ configuration to create a multicolor display [see Table 1]. To produce a transparent background with a colored “T”, a voltage of 1.5 V was applied to the WO_3_ electrode. Subsequently, the potential of the V_2_O_5_ electrode was varied to regulate the degree of ion insertion into the EC layer, thereby producing distinct colors for the “T”: 2.6 V for orange, 1.5 V for yellow-green, and 0.3 V for dark blue. Specifically, higher potential promotes deeper oxidation, resulting in an orange color, whereas moderate and lower potentials induce partial/full reduction of V^5+^ to V^4+^, leading to the appearance of yellow-green and dark blue colors, respectively. Alternatively, to create a blue background with a multicolor “T”, a voltage of 0.2 V was applied to the WO_3_ electrode, while the V_2_O_5_ electrode was adjusted to the same voltages as before to achieve various color states. This EC multicolor display is ideal for advertisements, digital billboards, and event exhibits, where customizable and eye-catching visuals are essential. Its key advantage lies in delivering vibrant, electronically controlled color transitions with low energy consumption, making it an efficient and versatile solution for a wide range of display applications.

The EC and energy-storage performances of the proposed devices were compared with previously reported three-electrode EC energy-storage systems, as summarized in Table 2. The WO_3-x_/Zn/WO_3-X_ device reported by Wu et al. exhibited relatively fast coloration and bleaching times of 4.8 and 3.6 s, respectively, with an optical modulation of 64% at 660 nm. However, its color transition was mainly limited to transparent and dark-blue states [22]. In comparison, Wang et al. reported a WO_3_/Al/Ti-V_2_O_5_ device with multiple color states and considerably higher energy-storage capacities of 302 and 631 mAh/m^2^ for WO_3_ and Ti-V_2_O_5_, respectively, although its coloration time was longer at 45 s [23]. Similarly, Song et al. demonstrated another Zn-based configuration, LaSVO/Zn/LaSVO, which enabled multiple color transitions with coloration and bleaching times of 24.9 and 5 s, respectively. However, the device exhibited a relatively low optical modulation of 10% at 531 nm, which was attributed to the color-overlay effect of the two EC thin films [14]. Furthermore, the V_2_O_5_/Zn/V_2_O_5_ configuration developed in this work exhibited an optical modulation of 48% at 633 nm and an energy-storage capacity of 185 mAh/m^2^, while enabling reversible color transitions from orange to yellow-green and blue. Meanwhile, the V_2_O_5_/Zn/WO_3_ configuration provided a higher optical modulation of 77.8% at 633 nm, with energy-storage capacities of 142 and 104.8 mAh/m^2^ for the WO_3_ and V_2_O_5_ electrodes, respectively, and enabled similar color transitions. Overall, the comparison indicates that different EC electrode combinations provide distinct balances between optical modulation, switching behavior, energy-storage capacity, and color versatility.

## 4. Conclusions

In conclusion, three-electrode EC energy storage devices with configurations of V_2_O_5_/Zn/V_2_O_5_ (energy status indicator) and V_2_O_5_/Zn/WO_3_ (energy storage window and multicolor display) were successfully developed, and their EC and electrical characteristics were thoroughly evaluated. The findings demonstrated that the V_2_O_5_/Zn/V_2_O_5_ configuration, with its great coloration depth across all color states and high energy storage capacity of 185 mAh/m^2^, was well-suited for energy status indicator applications where visible transitions were essential. In contrast, the V_2_O_5_/Zn/WO_3_ structure exhibited a superior optical contrast of 77.86%, a coloration efficiency of 46.24 cm^2^/C, and energy storage capacities of 104.8 mAh/m^2^ for V_2_O_5_ electrode and 142 mAh/m^2^ for WO_3_ electrode, respectively, making it ideal for application in multicolor energy storage windows. Furthermore, the V_2_O_5_/Zn/WO_3_ configuration also proved effective for multicolor display applications, enabling customizable color tuning of both EC electrodes. Overall, this study highlights the potential of three-electrode EC systems to surpass traditional EC devices, offering enhanced functionality through independent redox control, color overlay effects, and integrated energy storage capabilities. These advancements open new avenues for developing energy-efficient, multifunctional devices, with promising applications in smart windows, displays, and energy management systems. Nevertheless, long-term cycling stability and degradation behavior were not systematically investigated in this study and warrant further investigation to assess the long-term reliability of the proposed devices. Repeated ion insertion and extraction may induce structural and interfacial changes in the EC electrodes, potentially affecting their EC and electrical performance. In addition, electrolyte leakage and possible parasitic reactions, such as water decomposition, dendrite formation, and chloride oxidation, during prolonged operation may contribute to device degradation.

## Figures and Tables

**Table 1:** Multicolor display utilizing V_2_O_5_/Zn/WO_3_ structure.

	V_2_O_5_	Orange	Yellow-Green	Blue
WO_3_				
**Transparent**		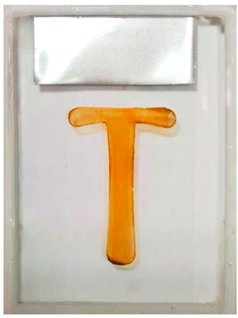	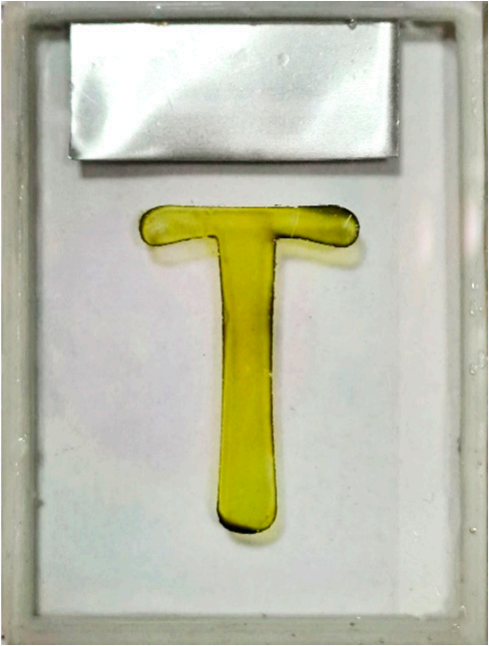	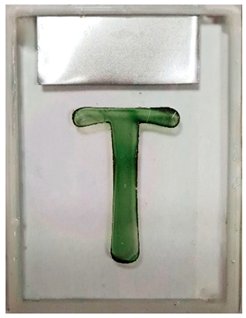
**Blue**		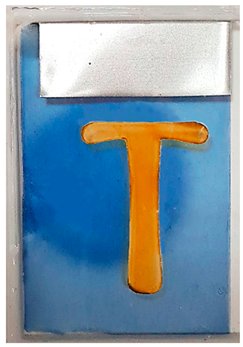	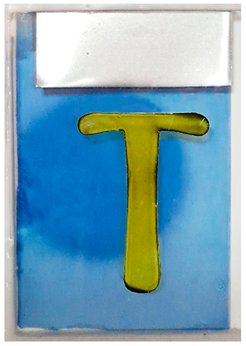	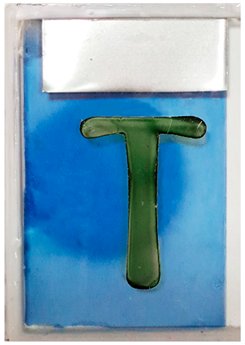

**Table 2:** Comparison of EC and energy-storage performance of three-electrode EC devices.

Ref	Device Configurations	Color/Bleaching Voltage (V)	Optical Modulation (%)	Energy Storage Capacity (mAh/m^2^)	Switching Time (s)	Color	Cycles
Wu et al. [22]	WO_3-x_/Zn/WO_3-X_	0/2	64% @ 660 nm	/	Tc: 4.8 Tb: 3.6	Transparent → Dark Blue	300
Wang et al. [23]	WO_3_/Al/Ti-V_2_O_5_	WO_3_: −1/+1 Ti-V_2_O_5_: −1.5/+2	60% @ 630 nm	WO_3_: 302 Ti-V_2_O_5_: 631	Tc: 45 Tb: -	Light yellow → transparent → light red → dark green → dark blue → black	500
Song et al. [14]	LaSVO/Zn/LaSVO	0.1/2.2	10% @ 531 nm	/	Tc: 24.9 Tb: 5	Green → chartreuse → brown → yellow → amber → orange	/
This work	V_2_O_5_/Zn/V_2_O_5_	0.3/2.6	48% @ 633 nm	185	/	Orange → yellow-green → blue	/
This work	V_2_O_5_/Zn/WO_3_	WO_3_: 0.2/1.5 V_2_O_5_: 0.3/2.6	77.8% @ 633 nm	WO_3_: 142 V_2_O_5_: 104.8	Tc: 33 Tb: 19	Orange → yellow-green → dark blue	/

## Data Availability

Data available on request of the authors.

## Abbreviations

The following abbreviations are used in this manuscript:

EC	Electrochromic
WO_3_	Tungsten trioxide
V_2_O_5_	Vanadium oxide
Zn	Zinc
H_2_O_2_	Hydrogen peroxide
ITO	Indium tin oxide
WCl_6_	Tungsten hexachloride
C_2_H_6_O	Absolute ethanol
CH_3_COOH	Glacial acetic acid
GCD	Galvanostatic charge/discharge
CA	Chronoamperometry
CV	Cyclic voltammetry
